# A targeted amplicon sequencing panel for cost-effective high-throughput genotyping of *Aedes aegypti*

**DOI:** 10.1186/s13071-026-07370-9

**Published:** 2026-04-15

**Authors:** Josquin Daron, Alicia Lecuyer, Laurence Ma, Pegah Marzooghi, Mallery I. Breban, Peter Kyrylos, I’ah Donovan-Banfield, Seth N. Redmond, Louis Lambrechts

**Affiliations:** 1 Insect-Virus Interactions Unit, Institut Pasteur, Université Paris Cité, CNRS UMR2000, Paris, France; 2 Biomics Core Facility, Center for Technological Resources and Research, Institut Pasteur, Université Paris Cité, Paris, France; 3 Canopy Clinical Research, Altona North, VIC Australia; 4 Department of Epidemiology of Microbial Diseases, Yale School of Public Health, New Haven, CT USA; 5 World Mosquito Program, Monash University, Melbourne, VIC Australia; 6 Research, Innovation and Commercialisation, University of Melbourne, Parkville, VIC Australia; 7 Department of Infection Biology and Microbiomes, Institute of Infection, Veterinary and Ecological Sciences, University of Liverpool, Liverpool, UK; 8 Department of Biochemistry, Cell and Systems Biology, Institute of Systems, Molecular and Integrative Biology, University of Liverpool, Liverpool, UK

**Keywords:** *Aedes aegypti*, Targeted amplicon sequencing, Population genomics, Genotyping, Population structure, Vector surveillance

## Abstract

**Background:**

The mosquito *Aedes aegypti* is the primary vector of several medically important arboviruses, including dengue, Zika, chikungunya, and yellow fever. Characterizing the genetic diversity of *Ae. aegypti* is essential to understand its evolutionary history and population dynamics and to evaluate vector control strategies. However, whole-genome sequencing (WGS) is often cost-prohibitive at scale due to the species’ large genome size. Reduced-representation approaches offer a cost-effective alternative, yet a standardized genome-wide marker set for population genetic studies of *Ae. aegypti* is lacking.

**Methods:**

We developed a targeted amplicon sequencing panel for cost-effective, high-throughput genotyping across 291 loci distributed throughout the *Ae. aegypti* genome. We evaluated its performance by comparing patterns of population structure and genetic admixture inferred from the amplicon sequences with those obtained from WGS data.

**Results:**

In silico analyses demonstrate that the amplicon panel reliably reproduces population structure patterns typically observed with WGS. It also effectively distinguishes among diverse laboratory colonies of *Ae. aegypti* and yields individual genetic admixture estimates consistent with WGS results.

**Conclusions:**

This targeted amplicon sequencing panel enables high-throughput genotyping at reduced cost and provides a practical alternative to WGS for population genetic and genomic surveillance studies. It should facilitate large-scale genotyping of *Ae. aegypti*, particularly in resource-limited settings.

**Graphical abstract:**

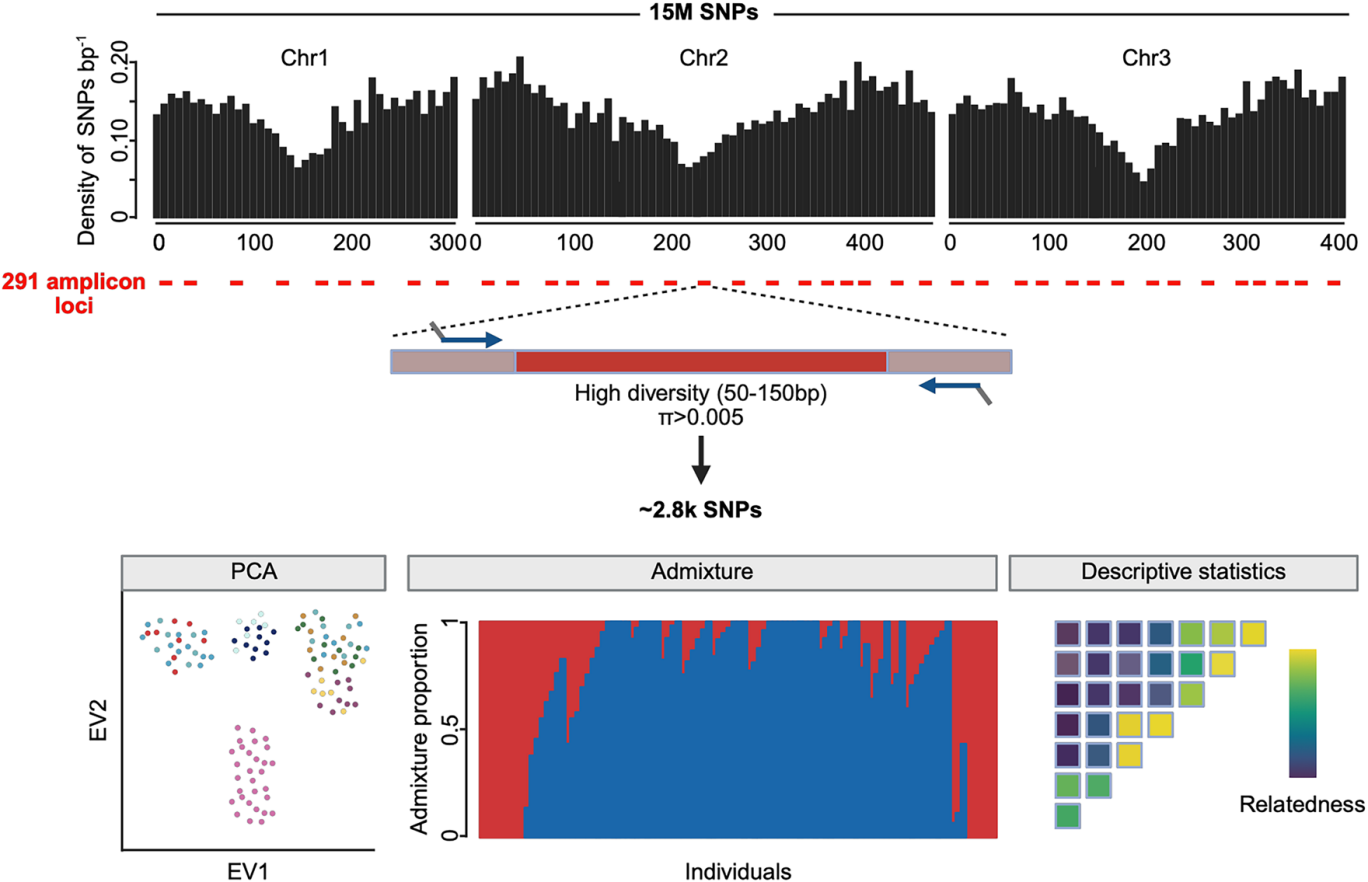

**Supplementary Information:**

The online version contains supplementary material available at 10.1186/s13071-026-07370-9.

## Background

The mosquito *Aedes aegypti* is the primary vector of multiple arthropod-borne viruses (arboviruses) of medical significance, such as dengue, Zika, chikungunya, and yellow fever [1]. Today, *Ae. aegypti* is prevalent throughout the tropics and subtropics, and its range is projected to expand further because of urbanization, connectivity, and climate change [2]. The species consists of two subspecies: *Aedes aegypti formosus* (*Aaf*), a generalist found in both forests and urban areas within its native African range, and the globally invasive *Aedes aegypti aegypti* (*Aaa*), a human specialist that thrives in urban environments [3, 4]. The human specialist *Aaa* evolved from generalist ancestors in West Africa approximately 5000 years ago [5], facilitating its global expansion over recent centuries. Its preference for human hosts, coupled with its higher vector competence, renders *Aaa* a highly effective transmitter of human arboviruses [6–9]. The dichotomy between *Aaa* and *Aaf* breaks down in some African locations where *Ae. aegypti* populations display patterns of genetic admixture between *Aaa* and *Aaf*, reflecting mixed genetic ancestry [8–12]. There is also extensive genetic diversity within each *Ae. aegypti* subspecies, particularly within *Aaf* [10, 12].

Population genetic studies of *Ae. aegypti* provide insights into the evolutionary pathways and genetic variations that have shaped this mosquito species over time. By examining its genetic structure, we can better understand how different environmental and biological factors influence population size, gene flow, and the spread of adaptive traits [5, 9, 10, 12]. By genetically monitoring mosquito populations, we can also examine the direct effect of vector control interventions by tracking changes in vector population size and dispersal rates [13, 14].

In the pre-genomic era, population genetic studies of *Ae. aegypti* mainly relied on small sets of genetic markers such as microsatellites. These simple sequence repeats are highly informative, but their number in the *Ae. aegypti* genome is limited [15, 16]. Moreover, they are prone to human error during scoring and require careful consideration for cross-study comparisons, complicating their use as high-throughput markers [17, 18]. With recent advances in high-throughput sequencing technologies, single-nucleotide polymorphisms (SNPs) and genotyping-by-sequencing methods have gained traction [19]. Unlike microsatellites, SNPs are abundant and straightforward, though they provide less information per marker as they are typically biallelic. However, their abundance allows large-scale screening to offset this limitation [20]. Despite declining costs of high-throughput sequencing technologies, whole-genome sequencing (WGS) remains cost-prohibitive for large-scale genotyping studies of *Ae. aegypti* because of its large genome size (1.3 Gb) and high repeat content (> 60%) [21–23].

In the last 2 decades, several alternative SNP-based genotyping methods to WGS have been developed, aiming to reduce costs by sequencing only a small portion of the genome, a concept known as reduced-representation approaches [19]. Restriction site-associated DNA sequencing (RAD-seq) has become a popular approach for identifying and screening SNPs [24] and has been successfully applied to *Ae. aegypti* [25–28]. Despite its usefulness, RAD-seq poses analytical challenges due to pervasive missing data [29, 30]. Building on RAD-seq data, a high-throughput genotyping chip with > 25,000 validated SNPs was developed for *Ae. aegypti* [31] and effectively used in studies of population structure and demographic inference [11, 32, 33]. This genotyping chip offers a more accurate and cost-effective alternative to low-coverage WGS (< 10 ×) for population genetic studies [34]. However, its reliance on a pre-selected set of SNPs introduces ascertainment bias [35]. Alternatively, low-coverage WGS has recently emerged as a cost-effective approach for genome-wide screening on a population scale, offering costs comparable to RAD-seq while providing comprehensive genomic data [36]. Nevertheless, this method presents a trade-off: it reduces coverage depth, which lowers confidence in individual genotype calls, and still entails relatively high library preparation costs and bioinformatic workload.

Targeted amplicon sequencing panels offer another valuable reduced-representation strategy for conducting cost-effective large-scale genotyping studies. These panels are designed to simultaneously amplify specific genomic loci using polymerase chain reaction (PCR). Barcoding of individual samples allows subsequent high-throughput sequencing of a multiplexed library. The resulting sequences are then compared with a reference genome to call SNPs and conduct various analyses. Furthermore, by focusing on sequenced regions containing multiple SNPs, one can determine “microhaplotypes” using phased short-read sequences [37]. Multi-allelic marker data from these loci significantly increase the power for relationship inference, such as estimating identity by descent [38]. These targeted amplicon panels have been developed for various applications, including vector species identification and pathogen detection [39–42], as well as for screening insecticide resistance [41, 43–45]. However, no targeted amplicon sequencing panel is currently available for population genetic studies of *Ae. aegypti*.

Here, we introduce a targeted amplicon sequencing approach that genotypes 291 loci across the *Ae. aegypti* genome. We provide evidence that this amplicon panel provides a cost-effective alternative to WGS for population genetic analyses, supporting its use for large-scale genotyping studies in resource-limited settings.

## Methods

### Amplicon panel design

Target loci were selected based on the AaegL5 assembly [23] and the Aaeg1200 genomes sequencing dataset [10]. Unless otherwise stated, all statistics were calculated using scikit-allel v1.3.1 [46]. Mean pairwise nucleotide diversity (*π*) was called in 50-base pair (bp) windows across the genome, and candidate target regions were identified as 50- to 150-bp high diversity regions (*π* > 0.005) flanked by low diversity regions (*π* < 0.0001) to design PCR primers. Regions with low diversity and known transposable elements identified in the AaegL5 assembly [23] were removed.

The remaining ~ 32,000 candidate regions were filtered to remove sites where high sequencing depth (> 20,000× in all samples) and/or low diversity (*π* < 0.02, < 25 SNPs per locus) might indicate a potential copy-number polymorphism. Low-depth samples (< 5000 ×) were removed to ensure high call rates. Target regions exhibiting strong selection signals (–2 < Tajima’s D < 2) [47] or inbreeding (Malecot’s ƒ < 0.25) [48] in either *Aaa* or *Aaf* populations were also removed. Out of 16,557 target regions that passed all filters, 10 sets of 350 potential loci each were pseudo-randomly selected using a gap-filling algorithm to optimize for both SNP and centimorgan (cM) distances [49].

Primer design was carried out at GT-seek LLC (https://gtseek.com) to obtain primers with suitable melting temperatures and target sizes for multiplex amplification [50]. Primers were designed to include a locus-specific sequence of 17–26 bp and an Illumina sequencing adapter of 31 bp (forward) or 34 bp (reverse). Potential dimer pairs were removed, and a set of 300 target loci was chosen to optimize for cM gap length. During optimization of the sequencing library preparation, nine primer pairs were subsequently excluded because of amplification artefacts. The final 291-locus panel had a median distance of 0.3 cM between markers. All primer sequences are provided in Table S1.

### DNA extraction

DNA was extracted from individual mosquitoes using the DNeasy 96 Blood & Tissue Kit (Qiagen), DNAzol DIRECT (Molecular Research Center, Inc.), or the Pat-Roman protocol, as previously described [51]. Both commercial kits were used following their manufacturer’s instructions. The Pat-Roman protocol is an inexpensive method relying on a homemade buffer to lyse the tissues, followed by a series of mixing, incubating, and centrifugation steps to isolate the DNA. Briefly, the mosquito was homogenized in a mixer mill (Precellys 24, Bertin Technologies) in Pat-Roman’s buffer (0.1 M NaCl, 0.2 M sucrose, 0.1 M Tris–Hcl pH 8.0, 0.05 M EDTA pH 8.0, 0.5% SDS, adjusted at pH 9.2). The lysate was incubated at 65 °C, and 8 M potassium acetate was added. After 30 min of incubation on ice, the lysate was transferred to a new tube and centrifuged for 15 min at 12,000 g. The supernatant was transferred to a fresh tube and mixed with 96% ethanol to precipitate DNA overnight at –20 °C. DNA was centrifuged for 30 min at 12,000 g, and the pellet was washed with ice-cold 75% ethanol, then with ice-cold 96% ethanol, each time followed by 5-min centrifugation at 12,000 g, and finally resuspended in TE buffer (Fisher Scientific).

### Library preparation and sequencing.

**Fig. 1:**
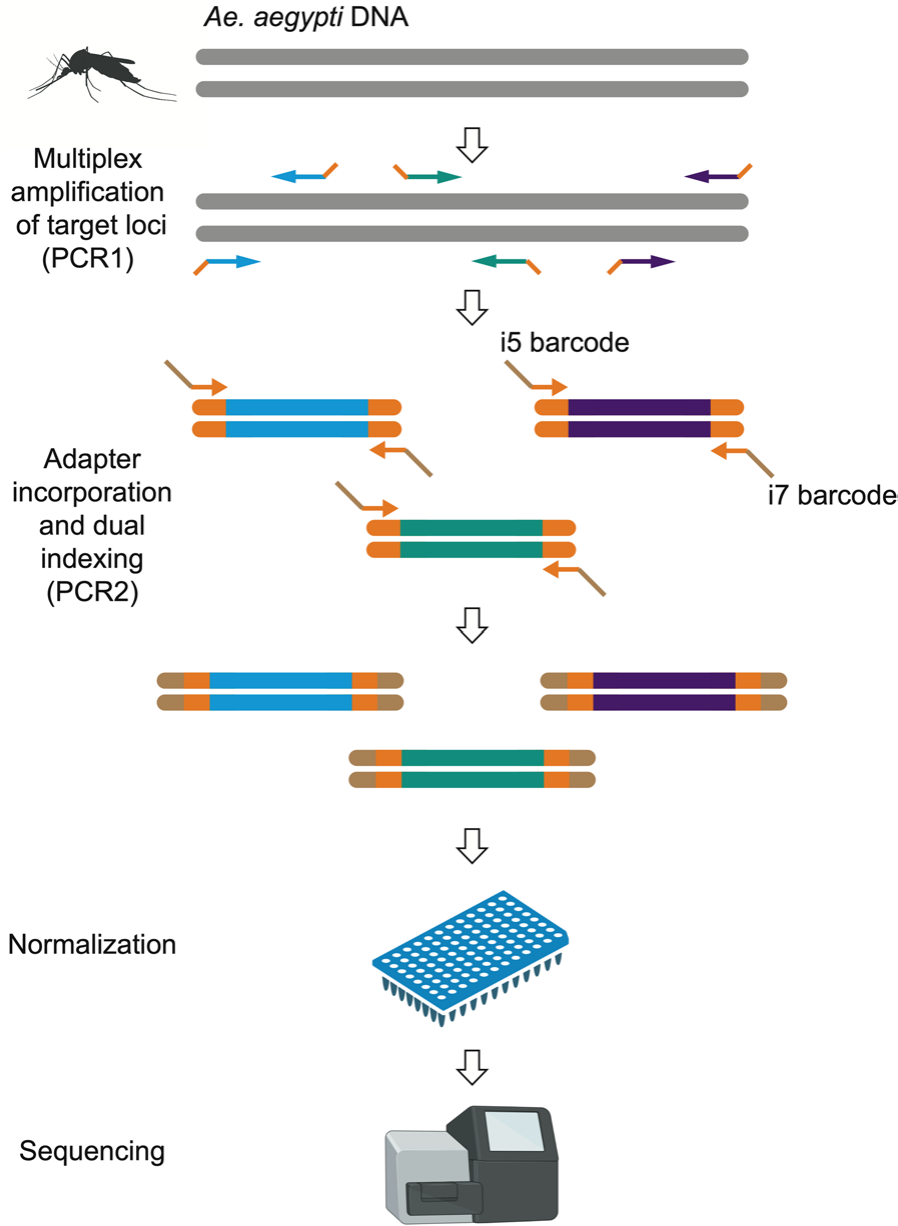
Targeted amplicon sequencing workflow. Amplicons were generated through two rounds of PCR. The first PCR (PCR1) involved amplifying 291 target loci simultaneously using a balanced primer pool, followed by a second PCR (PCR2) during which Illumina capture sites and unique dual indexes were added. The final products were normalized, pooled, and sequenced on an Illumina MiSeq 1st-generation platform

Amplicons were generated in two successive PCRs (Fig. 1). In the first PCR (PCR1), all 291 target loci were amplified simultaneously in a multiplex reaction, using a balanced primer pool. The PCR1 primers were initially resuspended in nuclease-free water to a concentration of 200 μM. To remove sequencing coverage extremes, each primer was then diluted individually using a balancing factor derived from initial test sequencing, as detailed in Table S1. Equal volumes of each diluted primer were combined in a single tube to create the balanced primer pool, resulting in an average concentration of ~ 50 nM per primer.

All PCR1 components were thawed at room temperature (20–25 °C), mixed well, and briefly centrifuged. For each sample, 2 μl of genomic DNA was mixed with 3.5 μl of Qiagen Plus Multiplex Master Mix 2 × and 3 μl of the balanced primer pool in a 96-well PCR plate (Fisher Scientific). The PCR1 cycling conditions were as follows. The reaction was initiated with a hot start at 95 °C for 15 min. A denaturation step was then conducted at 95 °C for 30 s, followed by a gradual ramp (5% or 0.2 °C/s) to 57 °C for a 2-min annealing step, and an extension step at 72 °C for 30 s, repeated for five cycles. This was followed by 15 cycles of denaturation at 95 °C for 30 s, annealing at 65 °C for 30 s, and extension at 72 °C for 30 s.

In the second PCR (PCR2), Illumina capture sites (P5 and P7) and unique dual indexes (i5 and i7) were appended to the PCR1 products by using PCR2 primers paired for combinatorial indexing (Table S1). The PCR1 products were diluted 20-fold in nuclease-free water, and 3 μl of the diluted PCR1 products was mixed in a 96-well PCR plate with 5 μl of Qiagen Plus Multiplex Master Mix 2 × and 1 μl of each PCR2 primer diluted at 2.5 μM. The PCR2 cycling conditions were as follows. The reaction was initiated with a hot start at 95 °C for 15 min. A denaturation step was then conducted at 95 °C for 10 s, followed by an annealing step at 65 °C for 30 s, and an extension step at 72 °C for 30 s, repeated for 15 cycles. A final extension was performed at 72 °C for 5 min.

PCR2 products were normalized using either a SequalPrep Normalization Plate Kit 96-well (Fisher Scientific) according to the manufacturer’s instructions, or manually to match the lowest concentration sample using a Qubit dsDNA HS Assay Kit (Invitrogen). Each normalized sample contributed 10 μl to a combined pool in a single tube. From this pool, 50 μl was mixed in a new tube with 40 μl of AMPure XP magnetic beads (Agencourt) at a ratio of 0.8 × and incubated at room temperature for 10 min. After placing the tube on a magnetic rack for an additional 10 min to allow the solution to settle, the cleared supernatant was removed and discarded. While still on the rack, 200 μl of freshly prepared 70% ethanol was added, incubated at room temperature for 30 s, and then discarded; this wash step was repeated once. After the second wash, the tube was taken off the rack and left open for 2 min at room temperature to slightly desiccate the beads. To elute the purified product, 15 μl of TE buffer (Fisher Scientific) was added to the dried beads, the mixture was thoroughly mixed, and the beads were returned to the magnetic rack. Once the solution cleared, the supernatant containing the purified library pool was collected and transferred to a new tube. The expected library size (218–305 bp) was verified using Bioanalyzer High Sensitivity DNA Kit (Agilent). Before purification, primer-adapter dimers often appeared as a secondary peak of ~ 170 bp, but they were generally eliminated by purification. If needed, additional removal could be achieved by performing a gel clean-up and/or slightly reducing the primer concentration. Sequencing was performed on a MiSeq first-generation platform (Illumina) using paired-end 2 × 150-bp reads, with either a MiSeq Reagent v2 Micro flow cell (yielding approximately 8 million reads) or MiSeq Reagent v2 Nano flow cell (yielding approximately 2 million reads).

### Processing of amplicon sequencing reads

The sequencing reads were initially demultiplexed by sample using the dual-index barcodes on the Illumina platform, resulting in one FASTQ file per sample. The raw reads were trimmed using Cutadapt v2.10 (parameters: − q 30 − m 50 –max-n 0) [52] and mapped to the AaegL5 reference genome assembly [23] using BWA-MEM v0.7.17 [53] with default parameters. PCR duplicates were removed using Picard Tools (http://broadinstitute.github.io/picard), and SNPs were called using GATK HaplotypeCaller v4.1.9.0 [54]. Only SNP variants were selected, and low-quality SNPs were excluded if they failed any of the following criteria: QD > 5, FS < 60, and ReadPosRankSum > –8. Genotypes with a genotype quality (GQ) ≥ 30 and sequencing depth ≥ 10 × were retained. To minimize missing data in the final SNP matrix, variants and individuals with > 10% missing data were discarded. An overview of this workflow is available from the GitHub repository at https://github.com/jdaron/ampliconSeq.

### Processing of WGS reads

The same genotyping procedure as for amplicon sequencing reads was applied to 656 publicly available WGS datasets for *Ae. aegypti* retrieved from NCBI bioprojects PRJNA602495 [9], PRJNA385349 [55], PRJNA882905 [8], PRJNA864744 [56], and PRJNA943178 [12]. The processing of these datasets followed the approach described above, with three adjustments made to account for the heterogeneous sequencing depth. First, the GQ threshold was relaxed to include genotypes with a GQ > 20 and a sequencing depth ≥ 5 ×. Second, variants with > 10% missing data and individuals with > 50% missing data were discarded. Third, the degree of relatedness among individuals (kinship coefficient) was estimated using plink v1.9 (parameter: –genome) [57], and samples with high relatedness (PI_HAT > 0.75) were excluded from subsequent population genetic analyses. This process ultimately yielded a final matrix of 493 individuals × 15 million SNPs.

### Putative off-target loci

To identify putative off-targets of the amplicon panel, each PCR1 primer pair was aligned against the reference genome using the primersearch tool from the EMBOSS suite [58], with the option -mismatchpercent 5, allowing for a single mismatch. The number of times each primer pair was mapped to the genome within a maximum distance of 300 bp was then calculated. Primer pairs producing more than one match in the genome were considered to have putative off-target loci.

### Population genetics

To explore population structure, SNPs in linkage disequilibrium were first removed by excluding those with an *r*^*2*^ > 0.01 within moving windows of 500 SNPs and a step size of 250 SNPs, using scikit-allel v1.3.3 [46]. The population genetic structure was visualized using principal component analysis (PCA) in scikit-allel. Individual genetic ancestry proportions were quantified using ADMIXTURE v1.3.0 [59], with the number of clusters (*K*) tested from 2 to 5. For the WGS dataset, ADMIXTURE was run 100 times for each *K* value, with each run using a random sample of 100,000 unlinked variants. The most likely number of ancestral populations (*K*) was determined using the cross-validation (CV) error rate [59]. The lowest CV error rate indicated that *K* = 2 ancestral populations was the most likely model.

## Results

### Genetic diversity of natural *Ae. aegypti* populations at amplicon loci

**Fig. 2:**
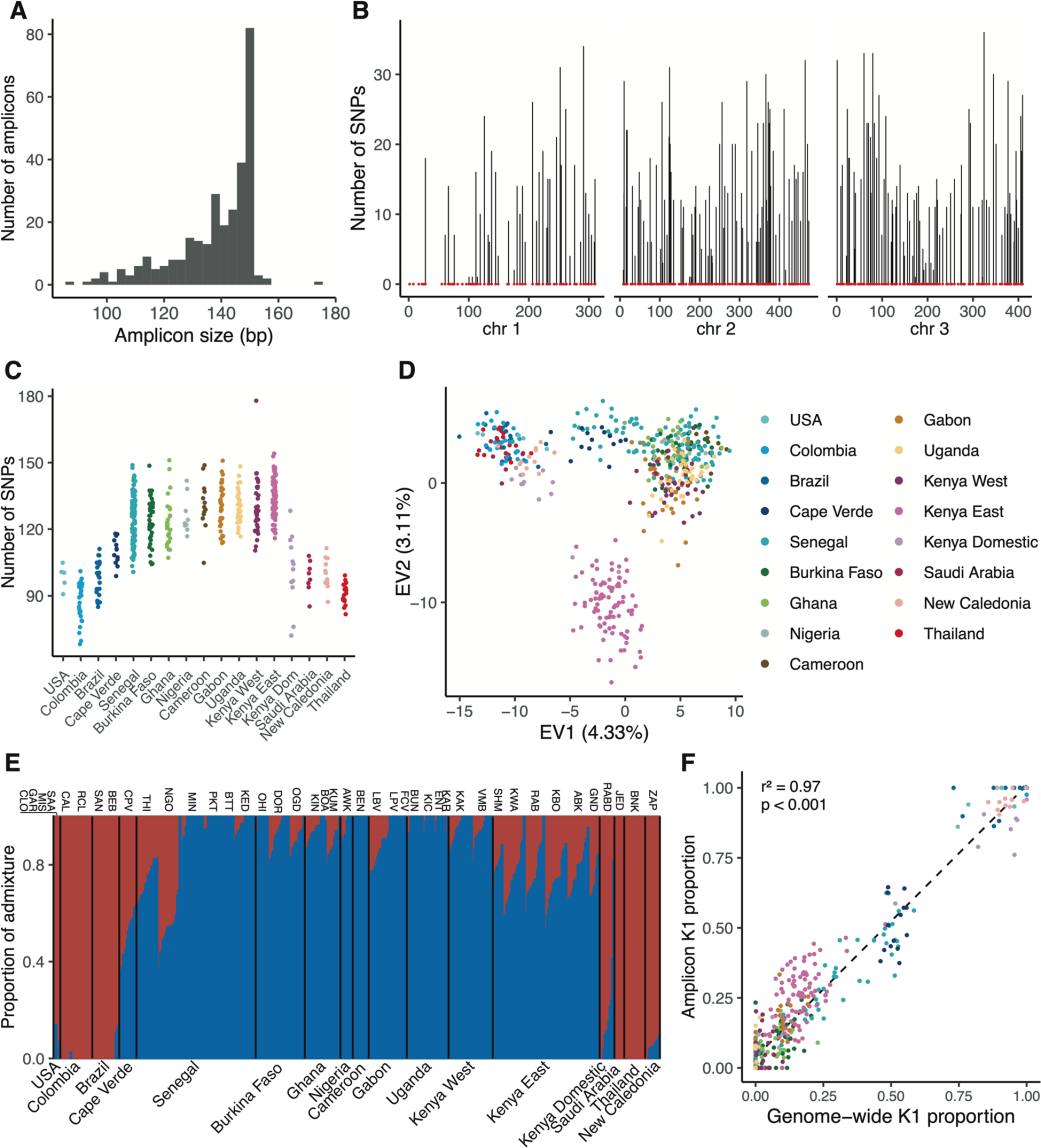
Global genetic diversity and population structure of natural *Aedes aegypti* populations inferred from amplicon loci. (**A**) Predicted amplicon size distribution. (**B**) Genomic distribution of amplicons (red dots) and number of SNPs identified at amplicon loci (vertical bars) along the three chromosomes (chr, shown in Mb) in natural populations worldwide. (**C**) Total number of SNPs identified per individual. (**D**) PCA of global genetic diversity at amplicon loci (EV = eigenvalue). (**E**) Barplot of admixture proportions per individual assuming two ancestry components (K = 2, with *Aaa* ancestry in red and *Aaf* ancestry in blue). (**F**) Correlation between the proportion of *Aaa* ancestry (K1) estimated from genome-wide data and from amplicon loci

We designed an amplicon panel of 291 loci collectively representing 41,423 bp (0.003% of the total *Ae. aegypti* genome size). The amplicon sizes range from 87 to 174 bp with a median of 143 bp (Fig. 2A). We first aimed to determine whether the genetic diversity captured by this amplicon panel recapitulates the population structure and diversity of *Ae. aegypti* observed on a genome-wide scale. We conducted a series of analyses using the amplicon sequences extracted from publicly available WGS data for *Ae. aegypti* [8, 9, 12, 55, 56]. From approximately 15 million SNPs identified, we extracted a set of 2826 SNPs across 493 individuals, present exclusively at the amplicon loci. These SNPs were evenly distributed across the genome, with a median of 12 SNPs per amplicon (Fig. 2B). We found that chromosomes 2 and 3 were particularly well covered, whereas the first 100 Mb of chromosome 1 exhibited the lowest density of suitable sites and SNPs. Our analysis revealed a higher number of SNPs in African *Ae. aegypti* populations than in those outside of Africa (Fig. 2C), consistent with earlier population genomic studies reporting greater genetic diversity in African populations [10, 12].

We next investigated the genetic structure of global populations using a set of unlinked SNPs. Principal component analysis (PCA) revealed three distinct clusters (Fig. 2D). African populations were separated into two clusters: one comprising all populations west of the Rift Valley and another comprising two Kenyan populations east of the Rift Valley. The third cluster consisted of populations from outside Africa, representing the invasive *Aaa* subspecies that expanded globally over the last few centuries [5].

Using Bayesian clustering, we characterized the genetic ancestry of the global samples, identifying two main ancestry components corresponding to *Aaa* (K1) and *Aaf* (K2) (Fig. 2E). As expected, several *Ae. aegypti* populations showed significant levels of admixture between K1 and K2. The proportion of K2 ancestry estimated from the amplicon loci was strongly correlated with the proportion estimated from WGS data (Pearson’s correlation coefficient, *r*^2^ = 0.97, *P* < 0.0001; Fig. 2F), demonstrating the ability of the amplicon panel to infer ancestry proportions.

### Genetic admixture in Cape Verde *Ae. aegypti*

**Fig. 3:**
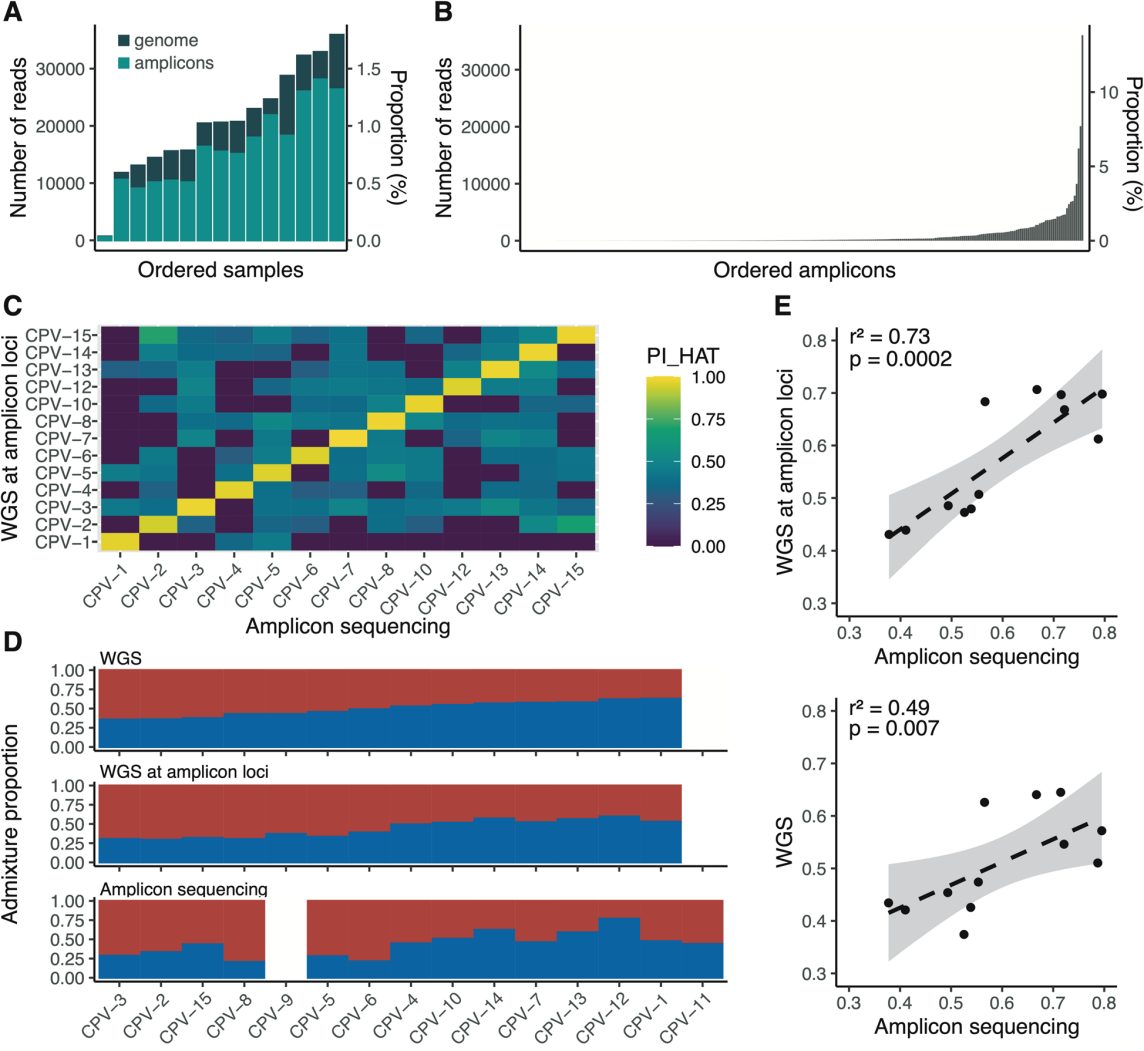
Admixture analysis of 15 wild-caught *Aedes aegypti* specimens from Cape Verde using amplicon sequencing vs. whole-genome sequencing. (**A**) Number of reads per Cape Verde (CPV) sample mapped to the amplicon loci (light green) or the AaegL5 assembly (dark green), respectively. (**B**) Number of reads uniquely mapping to each amplicon, ordered by increasing coverage. (**C**) Kinship (PI_HAT) matrix showing pairwise relatedness coefficients computed from amplicon loci genotyped using either WGS or amplicon sequencing. (**D**) Barplot of admixture proportions, assuming two ancestry components (K = 2), estimated from WGS data (top), polymorphisms at amplicon loci genotyped by WGS (middle), and polymorphisms at amplicon loci genotyped by amplicon sequencing (bottom). (**E**) Correlation between the proportion of *Aaa* ancestry estimated at amplicon loci using WGS and amplicon sequencing (top) and between genome-wide polymorphisms and amplicon sequencing (bottom)

Building on these results, we assessed the accuracy of the amplicon panel to infer mixed genetic ancestry. We genotyped 15 wild-caught *Ae. aegypti* samples from Cape Verde using the amplicon sequencing strategy, chosen because earlier WGS highlighted significant admixture between *Aaf* and *Aaa* (23% *Aaa* ancestry on average) in these samples [8]. Quality control showed successful mapping to amplicon loci for all but one sample and a relatively even read coverage across the other 14 samples (Fig. 3A). The distribution of read counts among amplicons was moderately skewed, with the most abundant amplicon accounting for ~ 13% of the reads (Fig. 3B).

For the 14 individuals that passed quality control, we genotyped 154 SNPs to evaluate genotype concordance between our amplicon sequencing data and WGS data using a relatedness analysis. Our pairwise comparisons showed high relatedness values between the same samples genotyped using the two different methods (Fig. 3C).

We next investigated the genetic ancestry of the Cape Verde samples by analyzing three sources of polymorphisms (Fig. 3D): (i) genome-wide polymorphisms identified in WGS data, (ii) polymorphisms identified at amplicon loci extracted from WGS data, and (iii) polymorphisms identified through our amplicon sequencing approach. We found a strong positive correlation (*r*^2^ = 0.73, *P* = 0.0002) between genetic ancestry proportions inferred from the amplicon loci using either amplicon sequencing or WGS data (Fig. 3E). This correlation was slightly weaker but still statistically significant (*r*^2^ = 0.49, *P* = 0.007) when comparing ancestry estimates from the amplicon loci to those derived from genome-wide polymorphisms (Fig. 3E). These results demonstrate that the amplicon panel can accurately estimate the proportions of *Aaf* and *Aaa* ancestry components in admixed samples while using far fewer markers than WGS, thus validating the cost-effectiveness of the amplicon sequencing approach for genetic ancestry inference.

### Genetic diversity of laboratory *Ae. aegypti* colonies

**Fig. 4:**
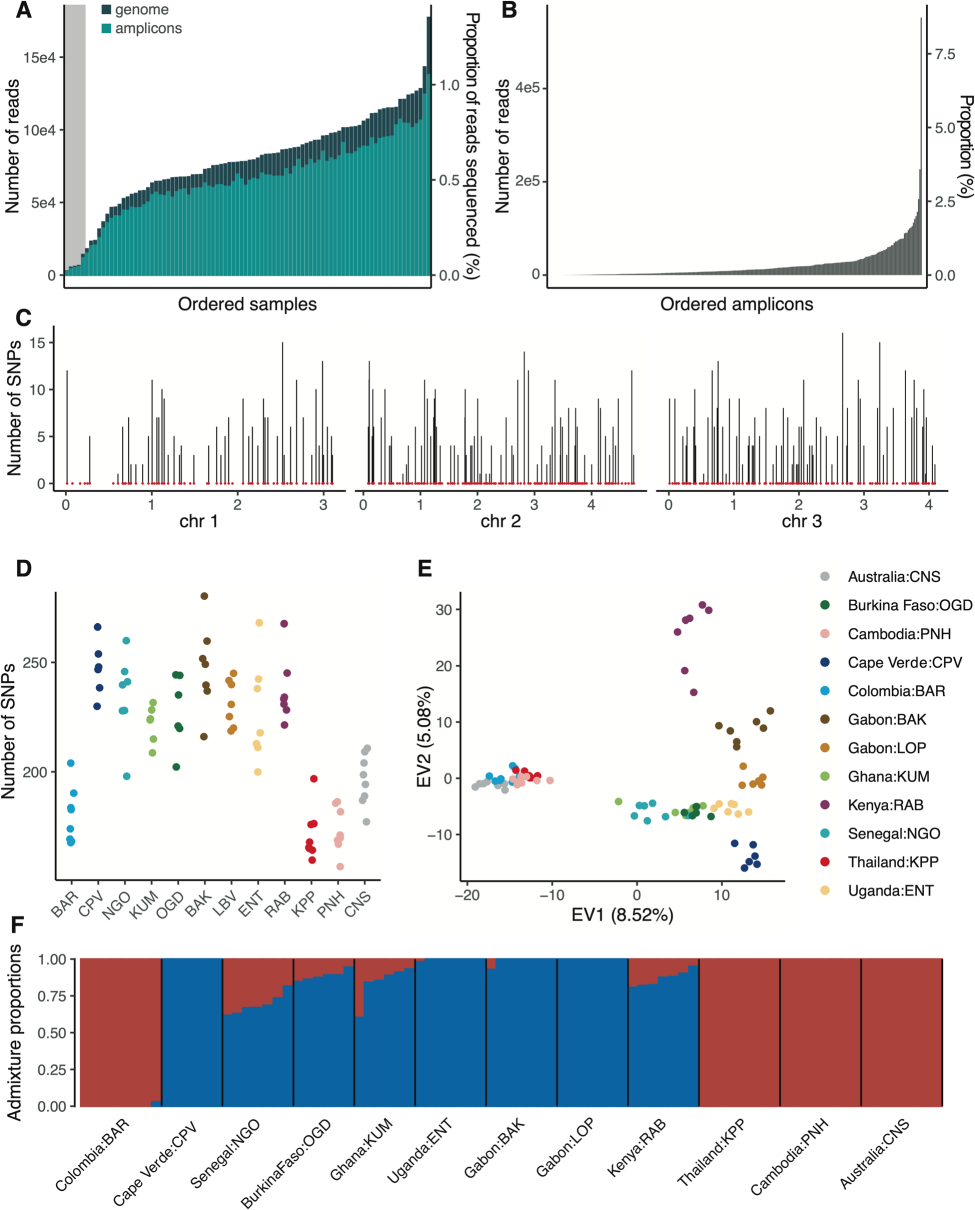
Genetic diversity and population structure of 12 laboratory colonies of *Aedes aegypti* determined by amplicon sequencing. Individual females from 12 colonies derived from field populations were analyzed after 7–29 generations (G) of laboratory colonization (BAR: Barranquilla, Colombia, G_19_; CPV: Praia, Cape Verde, G_7_; NGO: Ngoye, Senegal, G_17_; KUM: Kumasi, Ghana, G_15_; OGD: Ouagadougou, Burkina Faso, G_11_; BAK: Bakoumba, Gabon, G_26_; LOP: Lopé, Gabon, G_29_; ENT: Entebbe, Uganda, G_25_; RAB: Rabai, Kenya, G_18_; KPP: Kamphaeng Phet, Thailand, G_29_; PNH: Phnom Penh, Cambodia, G_26_; CNS: Cairns, Australia, G_23_). (**A**) Number of reads per sample mapped to the amplicon loci (light green) or the AaegL5 assembly (dark green), respectively. The gray shading indicates five samples that did not pass quality control and were excluded from subsequent analyses. (**B**) Number of reads uniquely mapping to each amplicon, ordered by increasing coverage. (**C**) Genomic distribution of amplicons (red dots) and number of SNPs identified at amplicon loci (vertical bars) along the three chromosomes (chr, shown in Mb) in the laboratory colonies. (**D**) Total number of SNPs identified per individual. (**E**) PCA of genetic diversity in the laboratory colonies at amplicon loci (EV = eigenvalue). (**F**) Barplot of admixture proportions per individual assuming two ancestry components (K = 2, with *Aaa* ancestry shown in red and *Aaf* ancestry shown in blue)

To validate the proof of concept for our amplicon panel, we expanded the range of samples under investigation. We genotyped a total of 90 individuals from 12 different field-derived *Ae. aegypti* colonies after 7–29 generations of laboratory colonization. We generated between 2352 and 137,943 reads per sample mapping to the amplicon loci (Fig. 4A). The read coverage per amplicon was relatively uniform, with no amplicons disproportionately represented (Fig. 4B). After removing five individuals presenting > 10% missing data, 1373 SNPs were genotyped across the remaining 85 individuals. These SNPs were evenly distributed across the genome (Fig. 4C). Consistent with earlier findings in natural populations of *Ae. aegypti* [10, 12], the number of SNPs per individual was higher in colonies originating from African populations than in those originating from non-African populations (Fig. 4D). PCA revealed clear clustering of individuals within colonies (Fig. 4E). Eigenvalue 1 (EV1) separated African from non-African colonies, while EV2 further differentiated African individuals along a West-to-Central-to-East African axis.

We inferred the genetic ancestry composition in the laboratory colonies and found admixture patterns that broadly matched expectations based on their geographic origins (Fig. 4F). Colonies originating from Africa showed a predominance of the generalist (*Aaf*) ancestry, with varying levels of admixture from the human-specialist (*Aaa*) ancestry, whereas non-African populations exhibited exclusive *Aaa* ancestry. Surprisingly, the colony originating from Cape Verde showed no signs of admixture, which was unexpected given the characteristics of the wild population it was derived from [8].

To assess the reproducibility of amplicon coverage across multiple sequencing runs, we prepared a new sequencing library with a subset of 75 individuals from the 12 laboratory colonies. Sequencing this new library produced between 2660 and 45,006 reads per sample mapping to the amplicon loci, which displayed adequate quality and mapping statistics overall (Figure S1). Amplicon coverage was highly consistent between the two sequencing runs, as demonstrated by a strong and significant correlation in the percentage of reads mapped to each amplicon (*r*^2^ = 0.81, *P* < 0.01; Figure S2). Our in silico analysis identified 60 primer pairs that were not specific to a single locus in the *Ae. aegypti* genome when allowing for a single mismatch. In both sequencing runs, amplicons with potential off-target loci did not show increased raw read coverage compared with the rest of the panel; however, they displayed significant enrichment in multi-mapping reads (Figure S3). Of the 60 amplicons with putative off-target loci, 92% and 86% were covered by multi-mapped reads in each run, respectively, whereas these proportions were 1.2% and 0.8% for the rest of the amplicons. To avoid any bias due to reads derived from off-target loci, all multi-mapping reads were excluded from downstream analyses.

### Cost-effectiveness of the amplicon panel

To guide future use of the panel, we aimed to determine the minimum sequencing depth necessary to accurately genotype the targeted polymorphisms without unnecessary excess. Using data from the sequencing run that yielded about 8 million reads for 90 samples, we performed a rarefaction analysis by computationally resampling from 1 to 20 million reads (Figure S4). We found that the proportion of genotyped amplicons (defined as those with at least 10 × depth of coverage) per sample increased asymptotically with the number of reads, with the curve plateauing at approximately 100,000 reads per sample. However, even at high sequencing depth, some variation in the proportion of genotyped amplicons persisted. This suggests that, for certain samples, there is insufficient template material to recover missing genotypes, and that further increasing sequencing depth will not resolve this limitation.

We also estimated the per-sample cost of our amplicon sequencing strategy, based on the current price list at our institutions (Table S2). These cost estimates are obviously subject to change and may also depend on pack size, special deals, and local taxes. We calculated the cost of generating 100,000 reads per sample, which we identified as the optimal read coverage based on the rarefaction analysis, using an 8-million read flow cell on an Illumina MiSeq first-generation platform. We estimated that at Institut Pasteur in France, the genotyping cost was 8.5 EUR per sample for library preparation and 13.6 EUR per sample overall, including the sequencing cost. At Yale University in the USA, the genotyping cost was approximately 10.1 USD per sample for library preparation and 27.8 USD per sample overall, including the sequencing cost. To help reduce the genotyping costs, we compared two commercial kits (Qiagen DNeasy Blood & Tissue and Molecular Research Center Inc. DNAzol DIRECT) and a low-cost, homemade protocol (called Pat-Roman) for DNA extraction. We compared the three DNA extraction methods based on the number of reads and SNPs genotyped for the same set of samples processed within the same library. Our analysis showed that the Pat-Roman protocol performed comparably to the Qiagen DNeasy Blood & Tissue kit, whereas the DNAzol DIRECT kit yielded poorer results (Figure S5). By implementing the Pat-Roman DNA extraction protocol and substituting plate normalization with manual normalization, we reduced the genotyping cost at Institut Pasteur to 4.6 EUR per sample for library preparation and 9.6 EUR per sample in total, including sequencing. At Yale University, the genotyping cost was reduced to 3.8 USD per sample for library preparation and 21.6 USD per sample in total, including sequencing.

## Discussion

In this study, we present a targeted amplicon sequencing strategy designed to enhance population genetic research on *Ae. aegypti*. This amplicon panel encompasses 291 loci throughout the *Ae. aegypti* genome and offers an accessible alternative to WGS, which is often cost-prohibitive because of the large size and high repeat content of the *Ae. aegypti* genome. By focusing on selectively neutral loci, the panel mirrors the genetic diversity and population structure typically inferred from WGS data, thus providing a valuable tool for large-scale genotyping studies. The amplicon panel will complement existing reduced-representation approaches, such as RAD-seq [25–28] and the commercially available *Ae. aegypti* SNP chip [31], without analytical challenges due to pervasive missing data [29, 30] or ascertainment bias [35].

Our results demonstrate that the amplicon panel effectively distinguishes genetic diversity and structure within and among *Ae. aegypti* populations globally. The panel’s robustness is supported by the consistency of our findings with known genomic patterns, such as higher SNP diversity in African populations and separation of global populations in three distinct PCA clusters [9, 10, 12]. However, the population-structure resolution of the amplicon panel was lower than that of genome-wide markers, likely because of the smaller number of variants and the shallow WGS coverage (mean 13.59 ×; range 0.55 × –35 ×). Furthermore, the panel’s ability to accurately estimate genetic ancestry components in naturally admixed populations highlights its utility in assessing admixture patterns in *Ae. aegypti*.

Our targeted amplicon sequencing panel provides a practical solution for genotyping *Ae. aegypti* at a fraction of the cost and resources required for WGS. We showed that the cost-effectiveness of the amplicon panel can be further improved by using a low-cost DNA extraction method and substituting plate normalization with manual normalization. While both modifications are more labor-intensive, they result in significant cost reductions, halving the library preparation costs and reducing the total cost per sample by about 25%. Costs could be further reduced by using minimally invasive methods, such as incubating a gently squashed mosquito in lysis buffer [40, 60], then directly incorporating the crude lysate into PCR, avoiding the need for homogenization, complex buffers, or precipitation steps.

Due to the reduced representation strategy of amplicon sequencing, there is a trade-off in genomic coverage, resulting in fewer SNPs compared to WGS, which may impact the resolution of certain population genetic analyses. Our rarefaction analysis identified 100,000 reads per sample as the optimal coverage point, yet some variability in amplicon coverage remains, potentially because of variations in the template material quality. Another limitation of the amplicon panel is the presence of multi-mapping reads, which must be excluded from downstream analyses. These multi-mapping reads presumably derive from off-target loci for a subset of 60 amplicons, which had lower uniquely mapping read coverage than the rest of the panel.

## Conclusion

Our newly developed amplicon panel provides a cost-effective solution for genotyping *Ae. aegypti* on a large scale, especially in resource-limited settings. Future enhancements could focus on expanding the panel to include more loci for vector species identification and detection of bacterial symbionts such as *Wolbachia* [39–42]. Additionally, these improvements could include screening for insecticide resistance [41, 43–45] and identification of large-scale chromosomal inversions using diagnostic SNPs [61]. Our targeted amplicon sequencing strategy opens the door for extensive population surveillance and vector control studies, enhancing our understanding of genetic dynamics, which in turn can inform public health strategies.

## Supplementary Information

Supplementary Material 1: Figure S1. Mapping statistics of the technical replicate run. (A) Number of reads per sample mapped to the amplicon loci (light green) or the AaegL5 assembly (dark green), respectively. (B) Number of reads uniquely mapping to each amplicon, ordered by increasing coverage

Supplementary Material 2: Figure S2. Amplicon sequencing reproducibility. Correlation between the percentage of reads mapped to each amplicon locus in the two replicate sequencing runs.

Supplementary Material 3: Figure S3: Visualization of amplicons with putative off-target amplification. The bottom panel shows the 291 amplicons, with vertical black lines indicating primer pairs that matched multiple sites in the genome. The top panel shows the number of reads mapped to each amplicon; gray bars represent uniquely mapped reads, while black lines represent multi-mapped reads. Kolmogorov-Smirnov and Wilcoxon tests were conducted to assess whether there was a difference in the number of mapped reads between amplicons with predicted off-target loci and the other amplicons.

Supplementary Material 4: Figure S4. Rarefaction analysis of the relationship between the amplicon coverage and sequencing effort. The graph shows the proportion of amplicons genotyped (> 10 × coverage) as a function of the read counts per sample. This analysis was performed by computationally resampling from 1 to 20 million reads using data from a sequencing run that yielded about 8 million reads. Different colors represent individual samples.

Supplementary Material 5: Figure S5. Comparison of three DNA extraction methods. Two commercial kits (Qiagen DNeasy Blood & Tissue and Molecular Research Center Inc. DNAzol DIRECT) and a low-cost homemade method (Pat-Roman) were compared based on the number of reads and SNPs genotyped from the same set of samples processed in the same library. The left panel shows the number of reads mapped to amplicon loci per sample, whereas the right panel displays the total number of SNPs identified. The data points are individual samples color-coded by colony, and the black vertical bar indicates the mean and 95% confidence interval. The *p*-values above the graphs were obtained using Wilcoxon test.

Supplementary Material 6: Table S1. Amplicon loci and primers. For each amplicon, the table provides the genomic positions, primer sequences, annealing temperature (Tm), and balancing factors.

Supplementary Material 7: Table S2. Cost estimates. The cost per sample was estimated based on the current price list at Institut Pasteur (France) and Yale University (US). The number of samples per Illumina MiSeq Micro flow cell (*n* = 80) was set to obtain 100,000 reads per sample, which we identified as the optimal read coverage based on the rarefaction analysis.

## Data Availability

Raw sequencing reads generated in this study have been deposited in the European Nucleotide Archive under accession no. PRJEB109091. All R scripts, bioinformatic scripts, and intermediate and final analysis files are available in an external data repository hosted on GitHub: https://github.com/jdaron/ampliconSeq.
