# Supplementary figures and images for: A targeted amplicon sequencing panel for cost-effective high-throughput genotyping of Aedes aegypti
Source: Parasit Vectors. 2026 Apr 15;19:227. doi: 10.1186/s13071-026-07370-9 (PMC13191845; doi:10.1186/s13071-026-07370-9)

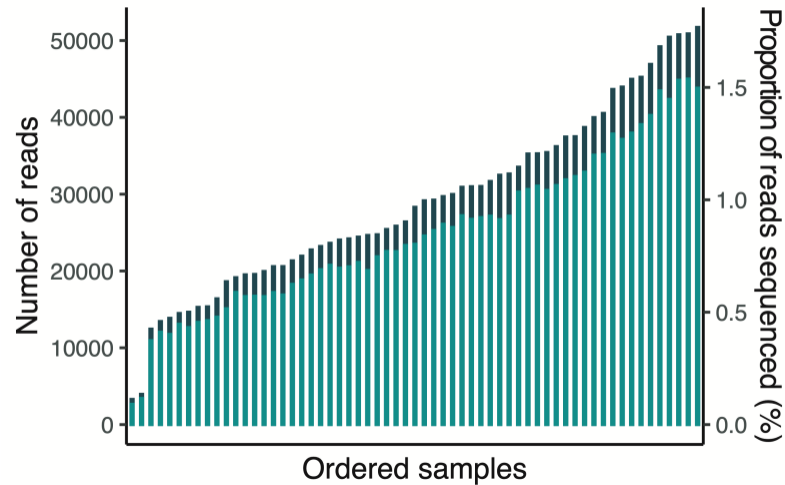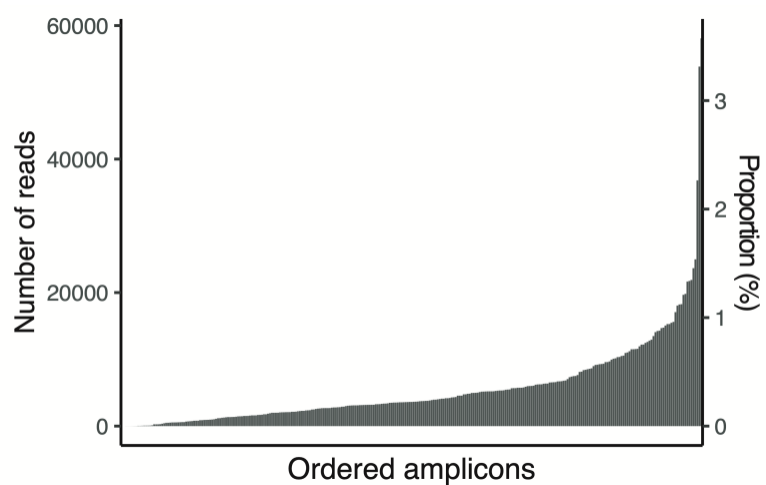

Supplement: Supplementary file 1 — Supplementary Material 1: Figure S1. Mapping statistics of the technical replicate run. (A) Number of reads per sample mapped to the amplicon loci (light green) or the AaegL5 assembly (dark green), respectively. (B) Number of reads uniquely mapping to each amplicon, ordered by increasing coverage [file 13071_2026_7370_MOESM1_ESM.pdf]

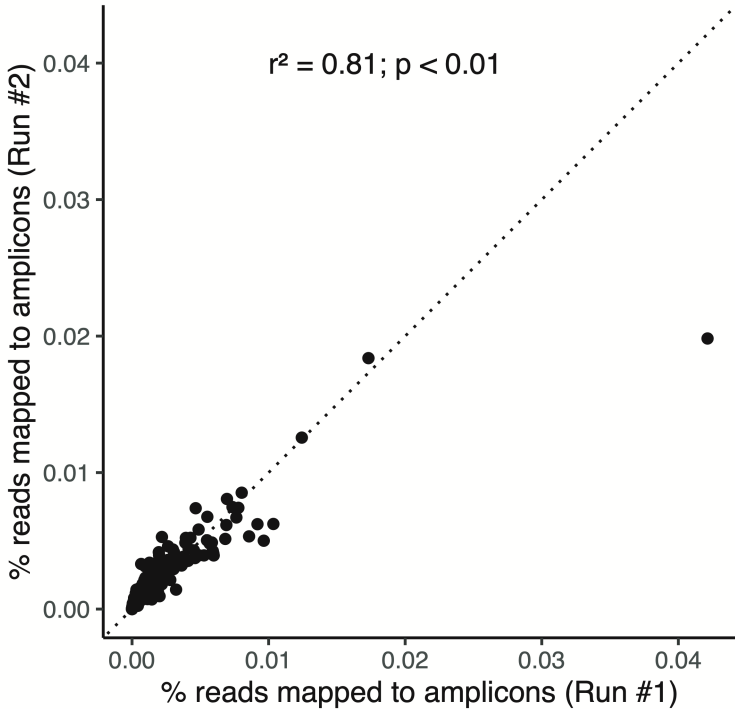

Supplement: Supplementary file 2 — Supplementary Material 2: Figure S2. Amplicon sequencing reproducibility. Correlation between the percentage of reads mapped to each amplicon locus in the two replicate sequencing runs. [file 13071_2026_7370_MOESM2_ESM.pdf]

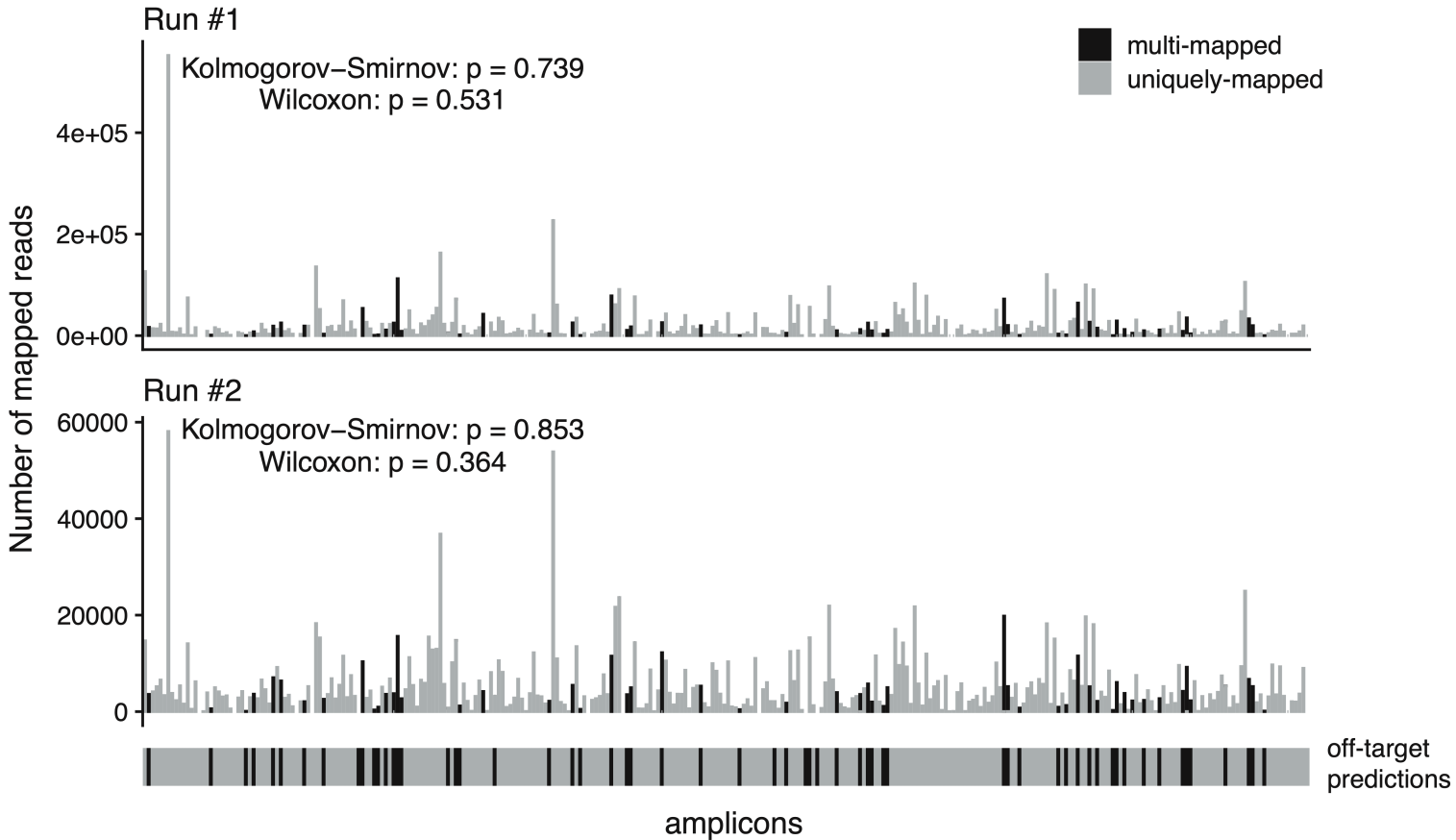

Supplement: Supplementary file 3 — Supplementary Material 3: Figure S3: Visualization of amplicons with putative off-target amplification. The bottom panel shows the 291 amplicons, with vertical black lines indicating primer pairs that matched multiple sites in the genome. The top panel shows the number of reads mapped to each amplicon; gray bars represent uniquely mapped reads, while black lines represent multi-mapped reads. Kolmogorov-Smirnov and Wilcoxon tests were conducted to assess whether there was a difference in the number of mapped reads between amplicons with predicted off-target loci and the other amplicons. [file 13071_2026_7370_MOESM3_ESM.pdf]

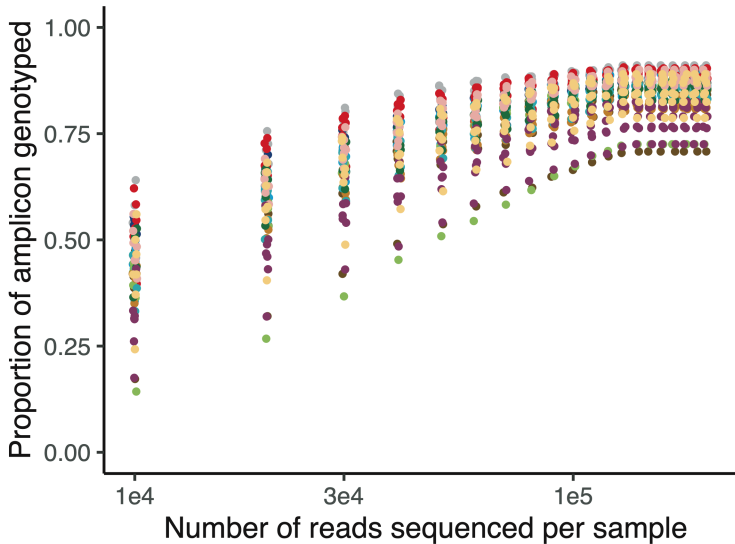

Supplement: Supplementary file 4 — Supplementary Material 4: Figure S4. Rarefaction analysis of the relationship between the amplicon coverage and sequencing effort. The graph shows the proportion of amplicons genotyped (> 10 × coverage) as a function of the read counts per sample. This analysis was performed by computationally resampling from 1 to 20 million reads using data from a sequencing run that yielded about 8 million reads. Different colors represent individual samples. [file 13071_2026_7370_MOESM4_ESM.pdf]

Number of reads

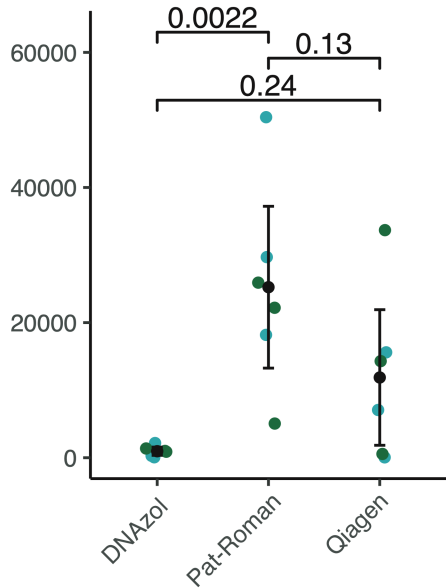

Number of SNPs

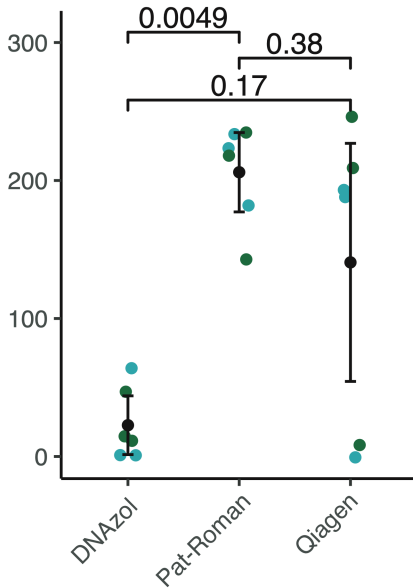

Lab colonies

- Senegal:NGO
- Burkina Faso:OGD

Supplement: Supplementary file 5 — Supplementary Material 5: Figure S5. Comparison of three DNA extraction methods. Two commercial kits (Qiagen DNeasy Blood & Tissue and Molecular Research Center Inc. DNAzol DIRECT) and a low-cost homemade method (Pat-Roman) were compared based on the number of reads and SNPs genotyped from the same set of samples processed in the same library. The left panel shows the number of reads mapped to amplicon loci per sample, whereas the right panel displays the total number of SNPs identified. The data points are individual samples color-coded by colony, and the black vertical bar indicates the mean and 95% confidence interval. The p-values above the graphs were obtained using Wilcoxon test. [file 13071_2026_7370_MOESM5_ESM.pdf]
